# Importance of the Side Chain at Position 296 of Antibody Fc in Interactions with FcγRIIIa and Other Fcγ Receptors

**DOI:** 10.1371/journal.pone.0140120

**Published:** 2015-10-07

**Authors:** Yuya Isoda, Hirokazu Yagi, Tadashi Satoh, Mami Shibata-Koyama, Kazuhiro Masuda, Mitsuo Satoh, Koichi Kato, Shigeru Iida

**Affiliations:** 1 Research Functions Unit, R&D Division, Kyowa Hakko Kirin Co., Ltd, Asahi-machi, Machida-shi, Tokyo, Japan; 2 Graduate School of Pharmaceutical Sciences, Nagoya City University, Tanabe-dori, Mizuho-ku, Nagoya, Japan; 3 JST, PRESTO, Tanabe-dori, Mizuho-ku, Nagoya, Japan; 4 Immunology & Allergy R&D Unit, R&D Division, Kyowa Hakko Kirin Co., Ltd, Asahi-machi, Machida-shi, Tokyo, Japan; 5 Institute for Molecular Science and Okazaki Institute for Integrative Bioscience, National Institutes of Natural Sciences, Higashiyama, Myodaiji, Okazaki, Aichi, Japan; 6 GLYENCE Co., Ltd., Chikusa, Chikusa-ku, Nagoya, Japan; 7 The Glycoscience Institute, Ochanomizu University, Ohtsuka, Bunkyo-ku, Tokyo, Japan; National Cancer Institute at Frederick, UNITED STATES

## Abstract

Antibody-dependent cellular cytotoxicity (ADCC) is an important effector function determining the clinical efficacy of therapeutic antibodies. Core fucose removal from *N*-glycans on the Fc portion of immunoglobulin G (IgG) improves the binding affinity for Fcγ receptor IIIa (FcγRIIIa) and dramatically enhances ADCC. Our previous structural analyses revealed that Tyr–296 of IgG1-Fc plays a critical role in the interaction with FcγRIIIa, particularly in the enhanced FcγRIIIa binding of nonfucosylated IgG1. However, the importance of the Tyr–296 residue in the antibody in the interaction with various Fcγ receptors has not yet been elucidated. To further clarify the biological importance of this residue, we established comprehensive Tyr–296 mutants as fucosylated and nonfucosylated anti-CD20 IgG1s rituximab variants and examined their binding to recombinant soluble human Fcγ receptors: shFcγRI, shFcγRIIa, shFcγRIIIa, and shFcγRIIIb. Some of the mutations affected the binding of antibody to not only shFcγRIIIa but also shFcγRIIa and shFcγRIIIb, suggesting that the Tyr–296 residue in the antibody was also involved in interactions with FcγRIIa and FcγRIIIb. For FcγRIIIa binding, almost all Tyr–296 variants showed lower binding affinities than the wild-type antibody, irrespective of their core fucosylation, particularly in Y296K and Y296P. Notably, only the Y296W mutant showed improved binding to FcγRIIIa. The 3.00 Å-resolution crystal structure of the nonfucosylated Y296W mutant in complex with shFcγRIIIa harboring two *N*-glycans revealed that the Tyr-to-Trp substitution increased the number of potential contact atoms in the complex, thus improving the binding of the antibody to shFcγRIIIa. The nonfucosylated Y296W mutant retained high ADCC activity, relative to the nonfucosylated wild-type IgG1, and showed greater binding affinity for FcγRIIa. Our data may improve our understanding of the biological importance of human IgG1-Fc Tyr–296 in interactions with various Fcγ receptors, and have applications in the modulation of the IgG1-Fc function of therapeutic antibodies.

## Introduction

To date, more than 30 monoclonal antibodies have been approved as drugs for the treatment of cancers, chronic diseases, and autoimmune diseases, and over 500 clinical trials investigating the application of monoclonal antibodies are currently ongoing. Within the last few years, several new antibodies have been approved as therapies, such as the anti-α4β7 integrin antibody vedolizumab, which was approved for the treatment of ulcerative colitis and Crohn’s disease in the USA in 2014 [1], and the anti-PD–1 antibodies nivolumab and pembrolizumab, which were approved for the treatment of malignant melanomas in Japan and the USA, respectively, in 2014 [2]. Antibody-dependent cellular cytotoxicity (ADCC), a lytic attack on antibody-targeted cells, is triggered by binding of Fcγ receptors (FcγRs) to the antibody constant region. Some clinical evidence with therapeutic antibodies, such as the anti-CD20 antibody rituximab, the anti-human epidermal growth factor receptor 2 (HER2) antibody trastuzumab, and the anti- epidermal growth factor receptor (EGFR) antibody cetuximab, has revealed that ADCC is one of the key mechanisms determining the clinical efficacy of these antibodies, although these antibodies also exhibit other anticancer functions (*e*.*g*., ligand neutralization, induction of apoptosis, and complement dependent cellular cytotoxicity) [3–9]. Human immunoglobulin G (IgG) has two *N*-linked oligosaccharide chains at conserved Asn–297 residues in each of the CH2 domains. The Fc *N*-glycans play an important role in the binding of Fcγ receptors and ADCC activity [10–14]. Core fucose removal from the Fc *N*-glycans had been reported to dramatically enhance ADCC activity via improved FcγRIIIa binding [12, 15–21]. Indeed, a nonfucosylated humanized anti-CC chemokine receptor 4 (CCR4) antibody, mogamulizumab, was approved in Japan in 2012 to treat relapsed/refractory CCR4-positive adult T-cell leukemia-lymphoma [22]. Most recently, obinutuzumab, a nonfucosylated humanized anti-CD20 antibody, also gained approval in the USA for the treatment of previously untreated chronic lymphocytic leukemia (CLL) [23]. Thus, ADCC enhancement technologies have shown clinical benefits in antibody therapeutics, and interest in resolving the mechanisms of mediating the FcγRIIIa affinity of nonfucosylated antibodies is growing. Recent structural analyses of the IgG1-Fc/shFcγRIIIa complex have revealed that the aromatic ring of Tyr–296 in nonfucosylated antibody is involved in interactions with *N*-glycans at Asn–162 and Lys–128 of FcγRIIIa through a hydrogen bond and van der Waals contacts [24, 25]. These findings demonstrate the structural importance of IgG1-Fc Tyr–296 in interactions with FcγRIIIa, particularly for the enhanced binding of nonfucosylated antibodies to FcγRIIIa. However, a detailed analysis of the importance of the Tyr–296 residue of the antibody in the interactions with various Fcγ receptors has not been reported. In this study, comprehensive Tyr–296 mutants were generated in the fucosylated and nonfucosylated forms of anti-CD20 chimeric IgG1s rituximab variants, and their binding affinities were determined for several soluble human activating Fcγ receptors, including shFcγRI, shFcγRIIa, shFcγRIIIa, and shFcγRIIIb. Our findings provide new insights into the biological significance of IgG1-Fc Tyr–296 and the potential for modulation of the effector function of therapeutic antibodies.

## Materials and Methods

### Cell lines

CHO/DG44 cells were obtained from Dr. Lawrence Chasin and Gail Urlaub Chasin (Columbia University, New York, NY) [26]. A *FUT8*-knockout CHO/DG44 was established for the production of nonfucosylated antibody, as previously reported [27]. The CD20^+^ B lymphoma cell line Raji (CCl–86) was purchased from the American Type Culture Collection (Manassas, VA).

### Blood donors

Blood donors were randomly selected from healthy volunteers registered at Tokyo Research Park (Kyowa Hakko Kirin, Co., Ltd, Tokyo). All donors provided written informed consent before the analyses.

### Construction, expression, and purification of antibodies

Fucosylated and nonfucosylated anti-CD20 chimeric IgG1s with a wild-type Fc region and amino acid substitutions in their Fc regions (Y296G, Y296A, Y296V, Y296L, Y296I, Y296P, Y296M, Y296F, Y296W, Y296S, Y296T, Y296N, Y296Q, Y296C, Y296D, Y296E, Y296H, Y296K, or Y296R; Eu numbering) were prepared as previously described [27, 28]. The cDNA encoding the wild-type Fc region of IgG1 was subcloned into the pBluescript SK (-) phagemid vector (Agilent Technologies, Tokyo). The plasmid was used as the template for polymerase chain reaction (PCR) with the appropriate mutant primers and a Quik-Change Multi Site-Directed Mutagenesis Kit (Stratagene, LaJolla, CA). The cDNA fragments were cloned into antibody expression vectors, and the constructed anti-CD20 antibody expression vectors were introduced into CHO/DG44 or *FUT8*-knockout CHO/DG44 to produce antibodies, as described previously [29]. Production and purification of IgG1s from the culture supernatant were performed as previously reported [27]. The purity of the isolated antibodies was examined by sodium dodecyl sulfate polyacrylamide gel electrophoresis (SDS-PAGE).

### Preparation of hexa-His-tagged recombinant soluble human Fc receptors (shFcγRs)

The shFcγRI, shFcγRIIa-134R, shFcγRIIIa-158V, shFcγRIIIa-158F, and shFcγRIIIb were prepared as described previously [17, 30–32]. For all shFcγRs, the transmembrane and intracellular domains were replaced by a hexa-His tag. The concentrations of purified proteins were measured by determining the absorbance at 280 nm, and their purities and molecular weights were confirmed by SDS-PAGE.

Bis-*N*-glycosylated shFcγRIIIa bearing oligosaccharides at both Asn–45 and Asn–162 was prepared by Asn-to-Gln mutation of the other three glycosylation sites (Asn–38, -74, and -169), as described previously [31], and was used for X-ray structural analysis.

### Analyses of antibody-derived *N*-linked oligosaccharides

For the glycosylation analyses, the isolated antibodies were boiled with SDS and 2-mercaptoethanol at 96°C for 3 min and digested by recombinant peptide-*N*-glycosidase F (PNGase F; Sigma-Aldrich, St. Louis, MO) with 5% Triton X–100 (Sigma-Aldrich).

The Fc *N*-linked oligosaccharides were prepared by digestion with recombinant peptide-*N*-glycosidase F (PNGase F; Sigma-Aldrich) and analyzed by matrix-assisted laser desorption/ionization time-of-flight mass spectrometry (MALDI-TOF MS) in the positive-ion mode, as described previously [30].

### Antibody binding to shFcγRs

The binding kinetics of chimeric anti-CD20 mutant antibodies for each shFcγR (shFcγRI, shFcγRIIa, shFcγRIIIa-158V, shFcγRIIIa-158F, and shFcγRIIIb) were analyzed by surface plasmon resonance (SPR) measurement using a T100 biosensor instrument and CM5 sensor chips (BIAcore; GE Healthcare, Pittsburgh, PA), as described previously [24]. Briefly, assays were performed with anti-tetra-His antibody-immobilized CM5 sensor chips using an Amine Coupling Kit (BIAcore). The individual hexa-His-tagged shFcγRs were captured by the immobilized anti-tetra-His antibodies at a flow rate of 5 μL/min. Antibodies were diluted in HBS-EP+ Buffer (BIAcore) at various concentrations (for shFcγRI and shFcγRIIIa-158V: from 4 to 267 nM; for shFcγRIIa, shFcγRIIIa-158F, and shFcγRIIIb: from 8 to 534 nM), and each diluted antibody was injected into the shFcγRs-coated sensor chip at a flow rate of 30 μL/min. The experiments were performed with HBS-EP+ as the running buffer at 25°C. The shFcγRs and antibodies bound to the sensor surface were removed by injecting 10 mM HCl. The data obtained by the injection of antibodies were corrected for the blank control prior to data analysis. The dissociation constant (*K*_D_) for each shFcγR was calculated by steady-state analysis using BIAcore T100 kinetic evaluation software (BIAcore).

### Antigen binding analysis

The antigen binding of anti-CD20 IgG1 rituximab variants to the CD20^+^ B lymphoma cell line Raji was measured by flow cytometry. Raji cells (5 × 10^5^ cells) were stained with 100 μg/mL of anti-CD20 IgG1 variants at 4°C for 30 min. Fluorescein isothiocyanate (FITC)-conjugated anti-human IgG (H+L) (R&D Systems, Minneapolis, MN) was used as the secondary reagent to detect cell-bound anti-CD20 IgG1 variants. The stained cells were analyzed using a BD FACSCanto II (BD Biosciences, San Jose, CA) [19].

### ADCC assay

The ADCC activity of anti-CD20 IgG1 rituximab variants was measured using the lactate dehydrogenase (LDH) release assay, described previously [33]. Human peripheral blood mononuclear cells (PBMCs) prepared from healthy donors using Lymphoprep (Axis Shield, Dundee) were used as effector cells, and Raji cells were used as target cells. The assays were conducted at an effector/target ratio of 25/1 in the presence of antibodies.

### Crystallization, X-ray data collection and structure determination of the nonfucosylated IgG1-Fc-Y296W mutant in complex with shFcγRIIIa

A binary complex formed between the nonfucosylated IgG1-Fc fragment with a Y296W mutation and bis-*N*-glycosylated shFcγRIIIa was purified by size-exclusion chromatography (Superose 12; GE Healthcare), as previously described [24]. The IgG1-Fc-Y296W/shFcγRIIIa complex (10 mg/mL) was crystallized in a buffer containing 12% PEG20,000, 0.1 M MES (pH 6.5), and 1% Zwittergent 3–08 (Hampton Research) using a sitting drop vapor diffusion method at 20°C. The obtained crystal was cryoprotected through soaking with a crystallization mother liquor containing 5–15% glycerol in a gradual manner (three steps with 3-min intervals). The diffraction dataset was collected using synchrotron radiation at NW12A of the Photon Factory (Tsukuba) and processed using HKL2000 software [34]. The crystal parameters of the binary complex are shown in Table 1.

**Table 1:** Data collection and refinement statistics for the IgG1-Fc-Y296W/shFcγRIIIa complex.

Parameter	IgG1-Fc- Y296W/shFcγRIIIa
**Crystallographic data**	
Space group	*P*4_1_2_1_2
Unit cell *a* / *b* / *c* (Å)	77.3 / 77.3 / 351.0
**Data processing statistics**	
Beam line	PF-AR NW12A
Wavelength (Å)	1.00000
Resolution (Å)	50–2.90 (2.95–2.90)
Total/unique reflections	164,773 / 24,829
Completeness (%)	98.4 (100.0)
*R*_merge_ (%)	9.2 (49.6)
*I* / σ (*I*)	30.8 (4.6)
**Refinement statistics**	
Resolution (Å)	20.0–3.00
*R*_work_ / *R*_free_ (%)	20.4 / 25.0
Number of non-hydrogen atoms	
Protein [Fc(A) / Fc(B) / FcR(C)]	1695 / 1714 / 1268
Water	26
Sugar [Fc(A) / Fc(B) / FcR(C)]	100 / 89 / 149
R.M.S. deviations from ideal	
Bond lengths (Å)	0.011
Bond angles (°)	1.57
Ramachandran plot (%)	
Favored	96.0
Allowed	4.0
Disallowed	0
Average *B*-factors (all atoms, Å^2^)	
Protein [Fc(A) / Fc(B) / FcR(C)]	61.1 / 53.0 / 75.3
Water	46.2
Sugar [Fc(A) / Fc(B) / FcR(C)]	74.8 / 67.4 / 103.1

The 3.00 Å-resolution crystal structure of IgG1-Fc-Y296W/shFcγRIIIa was solved by the molecular replacement method using the program MOLREP [35] with the crystal structure of wild-type IgG1-Fc complexed with shFcγRIIIa (PDB code: 3AY4) as a search model. Model building and refinement were conducted using COOT [36] and REFMAC5 [37], respectively. The stereochemical quality of the final model was evaluated using RAMPAGE [38]. The refinement statistics are summarized in Table 1. The molecular graphics were created using PyMOL (http://www.pymol.org/).

## Results

### Generation of fucosylated and nonfucosylated anti-CD20 IgG1 variants

**Fig 1:**
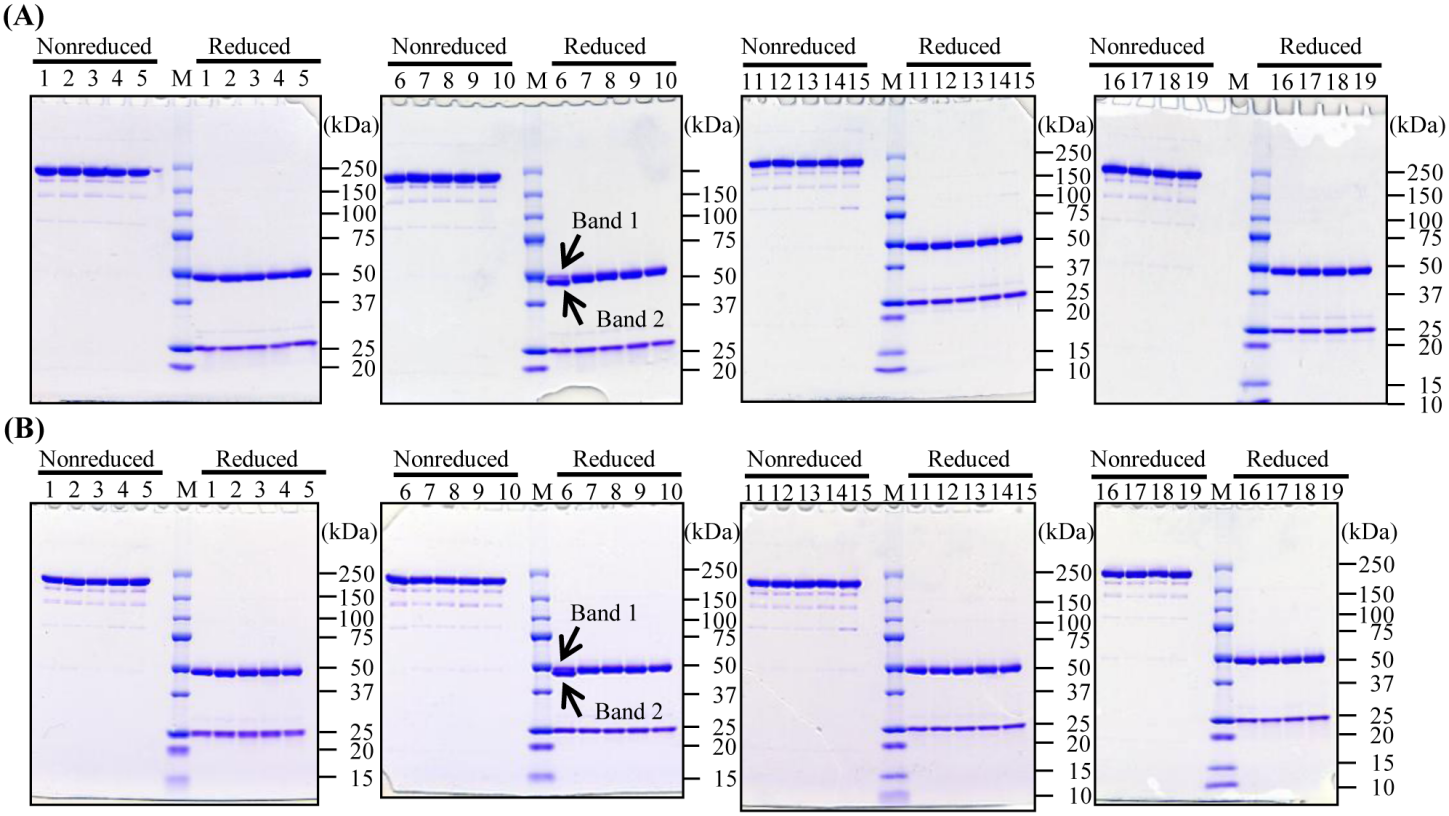
SDS-PAGE of the purified anti-CD20 IgG1 rituximab variants. The purified fucosylated (A) and nonfucosylated (B) anti-CD20 IgG1 variants were subjected to nonreducing and reducing 5–20% SDS-PAGE analyses. Lane M: molecular mass marker, lane 1: Y296G, lane 2: Y296E, lane 3: Y296L, lane 4: Y296F, lane 5: Y296D, lane 6: Y296P, lane 7: Y296H, lane 8: Y296W, lane 9: Y296S, lane 10: Y296R, lane 11: Y296N, lane12: Y296V, lane 13: Y296 A, lane 14: Y296I, lane 15: Y296C, lane 16: Y296T, lane 17: Y296K, lane 18: Y296M, and lane 19: Y296Q.

Serial mutants of the anti-CD20 chimeric IgG1 rituximab with amino acid substitutions in their Fc Tyr–296 residues were generated in fucosylated and nonfucosylated forms. In the purified Y296P mutant, two bands of the heavy chain were detected by reducing SDS-PAGE (Fig 1, lane 6, bands 1 and, 2). Band 2 was the major component and had a slightly lower molecular weight than band 1. PNGase F digestion revealed that the upper band of SDS-PAGE contained the *N*-glycan-deleted form (S1 Fig). Other anti-CD20 IgG1 variants showed the expected band sizes without any differences among them, although all variants showed some minor bands under nonreduced conditions (Fig 1, lanes 1–5 and 7–19). The fucose residue in the Fc *N*-glycans of anti-CD20 IgG1 variants was not detected in *FUT8*-knockout CHO/DG44-produced antibodies (Table 2). Unexpectedly, the oligosaccharide of the nonfucosylated Y296P variant could not be detected. In the CHO/DG44-produced antibodies, more than 97% of Fc *N*-glycans were fucosylated, with the exception of the Y296P variant bearing 16% high-mannose type Fc oligosaccharides.

**Table 2:** Oligosaccharide analysis of anti-CD20 IgG1 rituximab variants.

	Relative composition of oligosaccharides (%)							
	Nonfucosylated antibody				Fucosylated antibody			
Sample	High-Man	Fu(−)	Fu(+)	Total	High-Man	Fu(−)	Fu(+)	Total
Wild		100		100		2	98	100
Y296G		100		100			100	100
Y296E		100		100		3	97	100
Y296F		100		100			100	100
Y296P				N.D.	16		84	100
Y296H		100		100		2	98	100
Y296W		100		100		2	98	100
Y296R	3	97		100		3	97	100
Y296A	5	95		100		3	97	100
Y296K	5	95		100	2	3	95	100
Y296Q	3	97		100		2	98	100
Y296L	3	97		100			97	100
Y296D		100		100			100	100
Y296S		100		100		2	98	100
Y296N		100		100			100	100
Y296V		100		100			100	100
Y296I	2	98		100		3	97	100
Y296C	2	98		100			100	100
Y296T		100		100			100	100
Y296M		100		100			100	100

Each composition value is the relative amount of total complex-type oligosaccharides detected.

Fu(+): fucosylated complex-type sugar chains, Fu(−): nonfucosylated complex-type sugar chains, High-Man: high-mannose-type sugar chains, N.D.: not detected.

### FcγR binding profiles of anti-CD20 IgG1 variants

**Fig 2:**
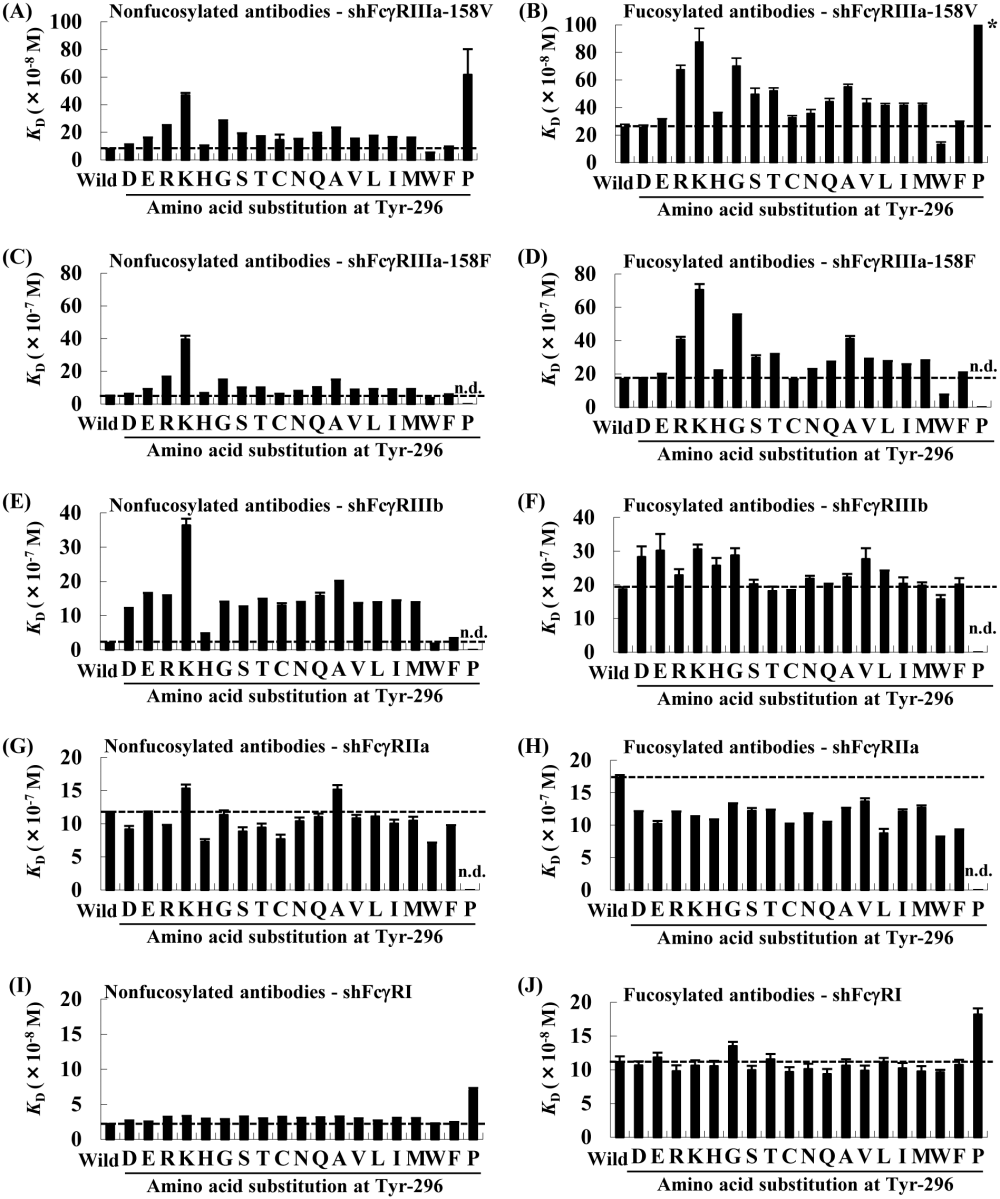
Binding affinities of anti-CD20 IgG1 rituximab variants for shFcRIIIa (V), shFcRIIIa (F), shFcRIIIb, shFcRIIa, and shFcRI. Binding affinities of the nonfucosylated antibodies (A, C, E, G, I) and fucosylated antibodies (B, D, F, H, J) for shFcRIIIa (V), shFcRIIIa (F), shFcRIIIb, shFcRIIa, and shFcRI were determined using Surface Plasmon Resonance(SPR) measurement. The mean *K*_D_ value (n = 3) is indicated on the Y axis; bars ± standard deviations (SDs). Dashed lines indicate the *K*_D_ value of the wild-type antibodies. *, the *K*_D_ value of Y296P was more than 100 × 10^−8^ M. n.d., not detected.

The binding kinetics of the anti-CD20 IgG1 variants to shFcγRI, shFcγRIIa, shFcγRIIIa-158V, shFcγRIIIa-158F, and shFcγRIIIb were estimated by surface plasmon resonance (SPR) measurement. Almost all nonfucosylated and fucosylated antibodies with the Tyr–296 mutation exhibited lower binding affinities for shFcγRIIIa-158V and shFcγRIIIa-158F than the wild-type antibody (Fig 2A–2D). In particular, Y296K and Y296P mutations significantly decreased the binding affinity of the nonfucosylated antibody for shFcγRIIIa compared with that of the fucosylated wild-type antibody. A relatively large loss of affinity was observed for Y296R, Y296G and Y296A mutants. On the other hand, only the Y296W mutant showed higher binding affinity for shFcγRIIIa than the parental antibody. The affinities of the fucosylated Y296W mutant for shFcγRIIIa-158V and -158F were increased by 1.93- and 2.20-fold, respectively, compared with that of the fucosylated wild-type antibody (fucosylated wild-type antibody: 25.7 ± 2.2 × 10^−8^ M versus fucosylated Y296W: 13.3 ± 1.8 × 10^−8^ M; fucosylated wild-type: 165 ± 3.7 × 10^−8^ M versus fucosylated Y296W: 74.9 ± 0.96 × 10^−8^ M, respectively) and the nonfucosylated Y296W mutant showed 1.46- and 1.36-fold lower *K*_D_ values for shFcγRIIIa-158V and -158F, respectively, than nonfucosylated wild-type antibody (nonfucosylated wild-type antibodies: 7.61 ± 0.44 × 10^−8^ M versus nonfucosylated Y296W: 5.23 ± 0.17 × 10^−8^ M; nonfucosylated wild-type antibodies: 49.9 ± 1.24 × 10^−8^ M versus nonfucosylated Y296W: 36.6 ± 0.95 × 10^−8^ M, respectively; Table 3). For the binding of shFcγRIIIb with nonfucosylated variants, only the Y296W mutant retained a high affinity comparable to that of the wild-type antibody, and a marked loss of affinity was observed with the Tyr–296 mutation in comparison with its fucosylated counterpart (Fig 2E and 2F). In the binding of shFcγRIIa, fucosylated Tyr–296 mutants, except for Y296P, showed greater affinity than fucosylated wild-type antibody. Y296W had the highest affinity among the tested fucosylated and nonfucosylated variants (Fig 2G and 2H). In contrast, shFcγRI binding was generally not affected by the Tyr–296 mutation, although the Y296P mutant exhibited an affinity that was several times lower than that of the wild-type antibody (Fig 2I and 2J).

**Table 3:** Binding affinities of anti-CD20 IgG1 rituximab variants to shFcγRs.

	Wild-type		Y296W		Y296A		Y296K	
Average *K*_D_ (×10^−8^ M) (*K*_D_ wild = 1)	Fu(+)	Fu(-)	Fu(+)	Fu(-)	Fu(+)	Fu(-)	Fu(+)	Fu(-)
FcγRIIIa(V)	25.7 ± 2.2 (1)	7.61 ± 0.44 (1)	13.3 ± 1.8 (1.93)	5.23 ± 0.17 (1.46)	54.9 ± 2.0 (0.47)	22.9 ± 0.59 (0.33)	87.6 ± 9.9 (0.29)	46.8 ± 1.6 (0.16)
FcγRIIIa(F)	165 ± 3.7 (1)	49.9 ± 1.2 (1)	74.9 ± 0.96 (2.2)	36.6 ± 0.95 (1.36)	411 ± 17 (0.4)	146 ± 3.6 (0.34)	704 ± 36 (0.23)	395 ± 23 (0.13)
FcγRIIIb	187 ± 6.7 (1)	20.2 ± 0.39 (1)	157 ± 13 (1.19)	17.9 ± 0.41 (1.13)	222 ± 11 (0.84)	198 ± 3.6 (0.1)	305 ± 14 (0.61)	364 ± 19 (0.06)
FcγRIIa	174± 3.4 (1)	116 ± 2.0 (1)	81.5 ± 0.98 (2.13)	69.2 ± 2.3 (1.68)	126 ± 1.2 (1.39)	151 ± 6.87 (0.77)	113 ± 1.2 (1.54)	153 ± 5.9 (0.76)
FcγRI	11.2 ± 0.81 (1)	10.4 ± 0.34 (1)	9.67 ± 0.34 (1.16)	8.1 ± 0.66 (1.28)	10.6 ± 0.93 (1.05)	10.1 ± 0.74 (1.03)	10.6 ± 0.78 (1.06)	10.5 ± 0.84 (0.99)

The values in parentheses indicated fold changes relative to wild-type. The mean *K*_D_ value (n = 3) is indicated on the Y axis; bars ± standard deviations (SDs). Fu(+): fucosylated; Fu(-): nonfucosylated.

### ADCC of anti-CD20 IgG1 mutants

To evaluate the influence of IgG1-Fc Tyr–296 mutations on the ADCC of the anti-CD20 IgG1 rituximab, the *in vitro* ADCC activities against Raji cells were measured using human peripheral blood mononuclear cells (PBMC) from two healthy donors as effector cells. Y296W, Y296K, and Y296A were selected as typical mutants, which showed the higher or dramatically lower affinity to the FcγRIIIa than wild-type. Flow cytometric analysis using Raji cells revealed that these antibody variants retained CD20 binding activity equal to that of the wild-type antibody (S2 Fig). The ADCC activities of antibodies were detected in both donors, although there was large variability between donor 1 and donor 2, which may have been related to differences in the *FCGRIIIa* genotype.

**Fig 3:**
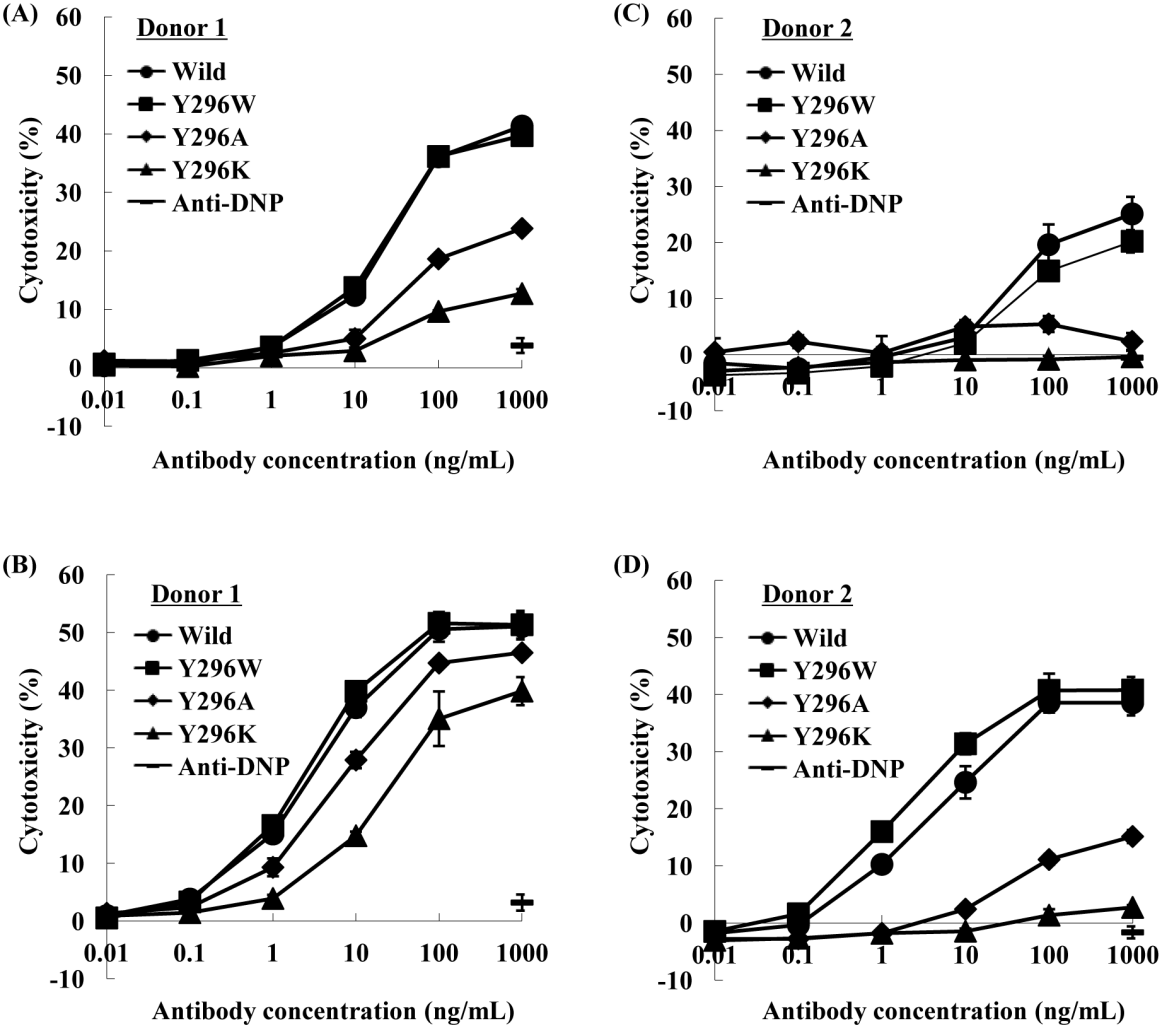
ADCC activity of anti-CD20 IgG1 variants. ADCC activities of anti-CD20 IgG1 rituximab fucosylated (A, C) or nonfucosylated (B, D) variants (WT: closed circle, Y296W: closed square, Y296A: closed diamond shape, Y296K: closed triangle, anti DNP antibody: bar) were measured by the LDH release method using CD20^+^ B-cell lymphoma cell line Raji cells as target cells and human PBMCs from two healthy donors (donors 1 [A, B] and donor 2 [C, D]) as effector cells at an E/T ratio of 20/1.

Irrespective of core fucosylation, the Y296A and Y296K mutants showed lower ADCC activities than the wild-type antibody (Fig 3), in the same order of their binding affinity to shFcγRIIIa (wild type > Y296A > Y296K; Table 3). The Y296W mutant having improved binding affinity to shFcRIIIa exhibited almost the same ADCC activity as the wild-type antibody (Fig 3).

### Structure of the nonfucosylated IgG1-Fc-Y296W mutant complexed with shFcγRIIIa

**Fig 4:**
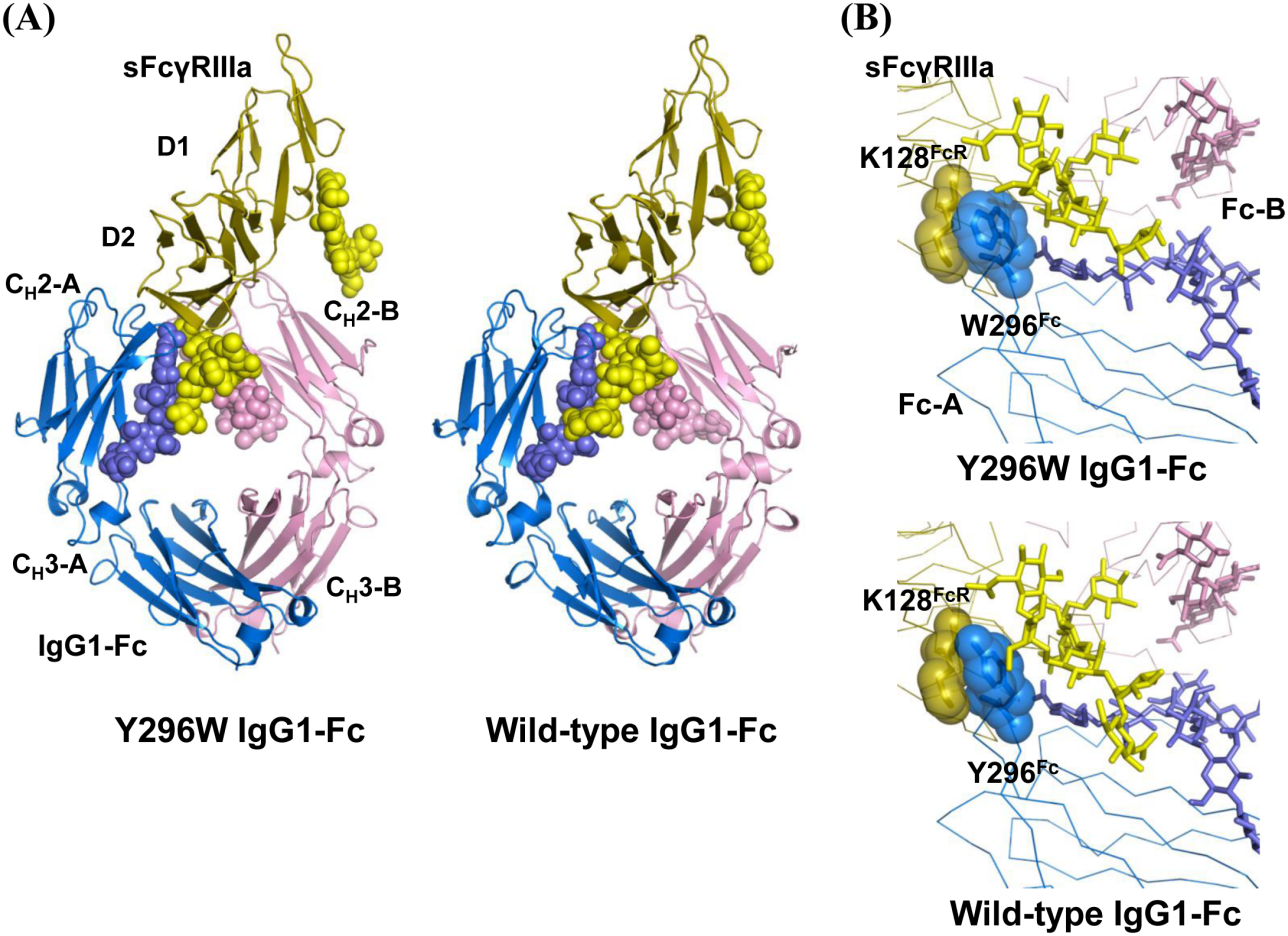
Structure of IgG1-Fc-Y296W complexed with shFcγRIIIa. (A) Overall structures of nonfucosylated Fc fragments in a complex with the bis-*N*-glycosylated soluble form of Fcγ receptor IIIa (shFcγRIIIa): left, Y296W Fc; right, wild-type Fc (PDB code: 3AY4). Chains A and B of the Fc fragment and shFcγRIIIa are colored marine, pink, and yellow, respectively. Carbohydrate residues are represented as spheres. (B) Close-up view of the interaction interface between Fc and shFcγRIIIa: upper, Y296W Fc; lower, wild-type Fc (PDB code: 3AY4). Carbohydrate residues are represented as sticks, and Lys–128 of sFcγRIIIa and Trp/Tyr–296 of Fc are represented as transparent spheres.

In order to understand the structural basis for the improved binding affinity due to the Tyr-to-Trp substitution at position 296 of the Fc portion, we determined the 3.00-Å-resolution crystal structure of the nonfucosylated IgG1-Fc Y296W mutant in complex with shFcγRIIIa harboring two *N*-glycosylations at Asn–45 and Asn–162 [31]. The overall structure of the mutated Fc/shFcγRIIIa complex was essentially identical to the previously reported crystal structures of the wild-type Fc complexed with the bis-*N*-glycosylated shFcγRIIIa mutant (RMSD = 0.21 Å for 581 Cα atoms; Fig 4A) [24, 25]. In the complex, the *N*-linked glycans displayed on both molecules exhibited well-defined electron densities (S3 Fig), showing unique carbohydrate-carbohydrate interactions between IgG1-Fc and shFcγRIIIa as previously described [24, 25].

In the interaction interface, the indole ring of the Trp–296 of Fc chain A was flipped out and made van der Waals contacts with Lys–128 and Man–4 of the Asn–162 *N*-glycan of shFcγRIIIa, as observed in the wild-type Fc (Tyr–296) complex (Fig 4B). Expectedly, the number of potential contact atoms in the complex with the Y296W mutant (indole group) is increased as compared with the wild-type counterpart (phenol group), thus contributing to the improved receptor-binding affinity of the Fc mutant.

## Discussion

Glycosylation of FcγRs is known to influence the affinities of these molecules for antibodies, and removal of core fucoses from *N*-linked oligosaccharides in the IgG1-Fc region can increase FcγRIIIa binding and dramatically enhance ADCC activity [12, 15–21]. In a previous study, we solved the structure of the complex formed between nonfucosylated IgG1-Fc and shFcγRIIIa with a minimal two *N*-glycans at Asn–45 and Asn–162 and showed that the Asn–162 *N*-glycan of shFcγRIIIa mediates the interaction with nonfucosylated Fc, thereby stabilizing the complex [24]. As for the glycoforms of FcγRIIIa, cell type- specific variation has been reported [39]. The recombinant FcγRIIIa used in this study was produced by CHO cells, and their glycoforms at Asn–162 were confirmed to have *N*-linked complex-type glycoforms [31]. Recently, Kawasaki et al. [40] reported the site-specific classification of *N*-linked oligosaccharides attached to the extracellular region of FcγRIIIb expressed in baby hamster kidney cells and identified their glycoforms as only complex-type at Asn–38, Asn–74, Asn–162, and Asn–169 and complex-type or high-mannose-type at Asn–45 and Asn–64. Taken together, these findings suggested that recombinant FcγRIIIa and FcγRIIIb produced by different cell lines have complex-type oligosaccharides as the major glycoforms at Asn–162. The Fc region and shFcγRIIIa have two binding modes depending on the orientation of the aromatic ring of the Tyr–296 residue of the Fc chain A. Core fucose depletion increases the occurrence of the active conformation of Tyr–296, thereby accelerating the formation of the high-affinity complex. Thus, Tyr–296 of the IgG1-Fc region plays an important role in interactions with shFcγRIIIa and enhancement of the binding affinity of nonfucosylated antibody for shFcγRIIIa. However, detailed analyses of comprehensive Tyr–296 mutants with a focus on the structural and functional importance of the Tyr–296 position in interactions with FcγRIIIa and other Fcγ receptors have not been reported. Our comprehensive binding analysis of Fc Tyr–296 mutants revealed in detail that Tyr–296 affected the binding of IgG1-Fc to not only FcγRIIIa but also FcγRIIa and FcγRIIIb.

IgG1-Fc Tyr–296 is located next to Asn–297, where the *N*-linked glycan is attached. *N*-glycosylation via oligosaccharyltransferase is known to be controlled by the specific conformation of the Asn residue within the consensus sequence Asn-X-Ser/Thr (where X is not Pro). Therefore, it was hypothesized that the amino acid substitution of Fc Tyr–296 in this study may somewhat affect Fc *N*-glycosylation. Indeed, Tyr to Pro substitutions resulted in a deglycosylated Fc and immature high-mannose type Fc oligosaccharides, possibly due to the restrained backbone conformation of Pro, which inhibits the access of oligosaccharyltransferase to Asn–297. These inhibitory effects of the adjacent proline on the *N*-linked glycosylation of the recombinant protein have also been reported in the production of recombinant human erythropoietin [41]. Importantly, the *N*-glycans at Asn–297 are crucial for Fc binding of FcγRs [10, 13, 14, 42] and are required for eliciting ADCC [11, 43]. In this study, insufficient *N*-glycosylation of Y296P also resulted in the marked reduction of binding to Fcγ receptors.

In contrast to Y296P, other Tyr–296 mutants showed binding affinity for Fcγ receptors. Notably, only the Y296W mutant showed increased binding affinity for FcγRIIIa through the additional Π-cation and Π-CH interactions via the indole group of Trp as compared with the binding affinity of the wild-type antibody. However, fucosylated Y296W never achieved the binding affinity of FcγRIIIa or ADCC activity of the nonfucosylated wild-type antibody. The ADCC activity of the nonfucosylated Y296W mutant was almost identical to that of nonfucosylated wild-type, as in the case of nonfucosylated IgG1s harboring triple Fc amino acid mutations that enable enhanced the binding to FcγRIIIa [33]. The ADCC and SPR assay results varied for fucosylated wild-type antibodies and the Y296W mutant. Although fucosylated Y296W improved the affinity for FcγRIIIa as compared with that of the fucosylated wild-type antibody, this mutant had almost the same ADCC activity as the fucosylated wild-type antibody. This may be explained by differences in FcγRIIIa binding kinetics of these antibodies. Masuda et al. (2007) revealed that *k*_ass_ but not *k*_dis_ of the antibody for FcγRIIIa binding is critical for enhancing ADCC activity [33]. In our experiment, the affinity was calculated as dissociation constant (*K*_D_) by steady-state analysis. Therefore, it is possible that fucosylated Y296W may improve *k*_dis_ rather than *k*_ass_. These results indicated that the enhanced FcγRIIIa binding of Y296W alone did not lead to ADCC improvement for wild-type IgG1 and that removal of the core fucose from Fc oligosaccharides was sufficient for maximizing the ADCC activity of IgG1.

In the interaction between Fc and FcγRIIIa, Tyr–296 forms a hydrogen bond and van der Waals contacts with the Asn–162 *N*-glycan and Lys–128 of FcγRIIIa, although the interactions are inhibited by the intramolecular contacts of Fc core fucose residues with the Tyr–296 tyrosine ring [24]. In this study, the fucosylated mutants, Y296A, Y296G, Y296K, and Y296R exhibited markedly reduced FcγRIIIa binding, and Y296A and Y296K showed diminished ADCC activity. Ala and Gly are fundamental amino acid residues whose side chains include only hydrogen or a methyl group, whereas Arg and Lys are basic amino acid residues with a positive charge. These characteristics of the amino acid residues may influence the contacts with the Asn–162 *N*-glycan or Lys–128 of FcγRIIIa, and reduce the affinity of Fc for FcγRIIIa. The same trends were also observed in the nonfucosylated counterparts; however, removal of the Fc core fucose markedly restored the ADCC of Y296A and Y296K mutants, possibly through the interaction between the Asn–162 *N*-glycan of FcγRIIIa and nonfucosylated Fc *N*-glycan [24, 25]. These findings suggested that each interaction with Fc Tyr–296 and the carbohydrate-carbohydrate contacts independently contributes to the high affinity of nonfucosylated IgG1 for FcγRIIIa and that carbohydrate-carbohydrate interactions can function even in the Fc exhibiting reduced FcγRIIIa binding because of the Tyr–296 mutation. Notably, the Tyr–296 mutants substituted with amino acid residues having aromatic rings showed improved or comparable binding affinities for FcγRIIIa as compared with that of the wild-type antibody. The Tyr to Phe mutation Y296F is known to be present in human IgG2, IgG3, and IgG4 subclasses, and Niwa et al. (2005) revealed that core fucose removal from IgG2/3/4 Fc-oligosaccharides can enhance the ADCC activity of these antibodies in a subclass-independent manner [19]. In this study, fucose depletion from the Fc-oligosaccharide of the Y296F mutant enhanced FcγRIIIa binding, as observed in wild-type Fc-Tyr–296. Therefore, IgG2/3/4-Fc Phe–296 was considered a key amino acid residue, corresponding to the IgG1-Fc Tyr–296, in the enhanced binding of nonfucosylated IgG2/3/4 to FcγRIIIa. Further analysis of IgG allotypes with amino acid variations at position 296, such as G3m14 (either Tyr–296 or Phe–296) and G3m15 (Tyr–296), will provide insights into the biological significance of Phe–296 on the ADCC and other biological activities of IgG subclasses. The binding profiles of the Tyr–296 mutants for FcγRIIIb were similar to the binding profiles for FcγRIIIa, including the high binding affinity of the Y296W mutant. In contrast to FcγRIIIa binding, the nonfucosylated form showed a larger fold-decrease in affinity due to the amino acid substitution than its fucosylated counterpart. These findings suggested that the Fc 296 position was more important for the binding of nonfucosylated IgG1 to FcγRIIIb than for the binding of fucosylated IgG1.

The effects of the Tyr–296 mutation on the binding of FcγRI and FcγRIIa were different from those observed for FcγRIIIa. For FcγRI, unlike FcγRIIIa, only the amino acid substitution of Pro at the Tyr–296 position affected the interaction between the antibody and FcγRI. FcγRI has been reported to be a high-affinity receptor, and the FG-loop with its conserved one-residue deletion is critical for this high affinity [44]. Taken together with the results of this study, these finding suggested that the FG-loop may be the main contributor to the interactions between IgG1 and FcγRI.

FcγRIIa lacks an *N*-glycan corresponding to the Asn–162 of FcγRIIIa, and does not form *N*-glycan (Asn–64, Asn–145)-mediated interactions with IgG1-Fc [45]. The core fucose of the Fc oligosaccharide is believed to be closely apposed to the interaction sites between Fc and FcγRIIa-HR, comprising Lys–128 and Ser–129 of Fc, and Phe–132 and Arg–134 of the receptor. Removal of the fucose from the IgG1-Fc oligosaccharide has been shown to slightly increase binding to FcγRIIa-HR [46], which was also reproduced in this study (Table 3). As in the case of FcγRIIIa, FcγRIIa binding was reduced by Y296A and Y296K and improved by Y296W in the nonfucosylated form. However, the effects of these mutations on FcγRIIa binding were not as remarkable as those for FcγRIIIa. Although the 296 position of nonfucosylated Fc interacts with FcγRIIa, its contribution is thought to be limited because the additional binding mode via the receptor’s *N*-glycan, corresponding to Asn–162 of FcγRIIIa [24, 25], is not involved in FcγRIIa binding. In their fucosylated forms, Tyr–296 mutants, with the exception of Y296P, showed improved Fc binding affinity for FcγRIIa. The inhibitory effects of Tyr–296 on FcγRIIa binding were thought to be caused by intramolecular contacts with Fc core fucose [45] and reduced by the Tyr–296 mutation, creating additional interactions with Lys–128 and Ser–129, or Phe–132 and Arg–134 of FcγRIIa.

Taken together, our data showed that the Y296W mutant had a unique FcγR-binding profile compared with those of the other mutants, with improved binding for FcγRIIIa-158F (V), FcγRIIIb, and FcγRIIa-HR, irrespective of the Fc core fucosylation. Moreover, binding for FcγRI was comparable to that of the wild-type antibody. In terms of the biological significance of Y296W, the nonfucosylated form retained maximal ADCC activity. However, other activities, such as binding to the inhibitory receptor FcγRIIb [47], FcγR-mediated antibody-dependent cell-mediated phagocytosis (ADCP), ROS/cytokine production, antigen presentation [48], and neonatal Fc receptor-mediated pharmacokinetics [49], should also be evaluated in future studies in order to determine the potential for nonfucosylated Y296W to serve as a therapeutic antibody.

In summary, this study demonstrated the biological importance of human IgG1-Fc Tyr–296 for interactions with various FcγRs by protein engineering and X-ray structural analysis. Additionally, the unique binding profile of the Y296W mutant to FcγRs was identified as a potential format for ADCC-based therapeutic antibodies. Our results provide new information on FcγR-mediated antibody function and offer new clues for designing and engineering therapeutic antibodies with improved efficacy.

## Accession Numbers

The coordinate and structural factors of the crystal structure of the IgG1-Fc-Y296W/shFcγRIIIa complex have been deposited in the Protein Data Bank under accession number 5BW7.

## Supporting Information

S1 Fig Reducing SDS-PAGE of the anti-CD20 IgG1 rituximab variants digested by PNGaseF. The fucosylated and nonfucosylated anti-CD20 IgG1 variants that were digested by PNGaseF were subjected to reducing 5–20% SDS-PAGE analyses. Lane M: molecular mass marker, lane 1: fucosylated Y296P without digestion, lane 2: fucosylated Y296P with digestion, lane 3: fucosylated wild-type without digestion, lane 4: fucosylated wild-type with digestion, lane 5: nonfucosylated Y296P without digestion, lane 6: nonfucosylated Y296P with digestion, lane 7: nonfucosylated wild-type without digestion, lane 8: nonfucosylated wild-type with digestion.
(TIF)

S2 Fig Antigen binding activity of purified anti-CD20 IgG1 rituximab variants to CD20^+^ B-cell lymphoma cell line Raji cells. Rituximab variants binding to Raji cells were measured by flow cytometry. Cells were stained with 1 μg/mL anti-CD20 IgG1 rituximab variants (filled histograms) or staining buffer alone (blank histograms) at 4°C for 30 min, followed by staining with a detecting antibody (FITC-conjugated anti-human IgG antibody) at 4°C for 30 min.
(TIF)

S3 Fig Close-up view of the interaction interface between IgG1-Fc Y296W and shFcγRIIIa. The *F*_o_-*F*_c_ electron density map of *N*-glycans, Trp–296 (IgG1-Fc chain A), and Lys–128 (FcγRIIIa) contoured at 1.5 σ.
(TIF)
