# Supplementary figures and images for: Importance of the Side Chain at Position 296 of Antibody Fc in Interactions with FcγRIIIa and Other Fcγ Receptors
Source: PLoS One. 2015 Oct 7;10(10):e0140120. doi: 10.1371/journal.pone.0140120 (PMC4596520; doi:10.1371/journal.pone.0140120)

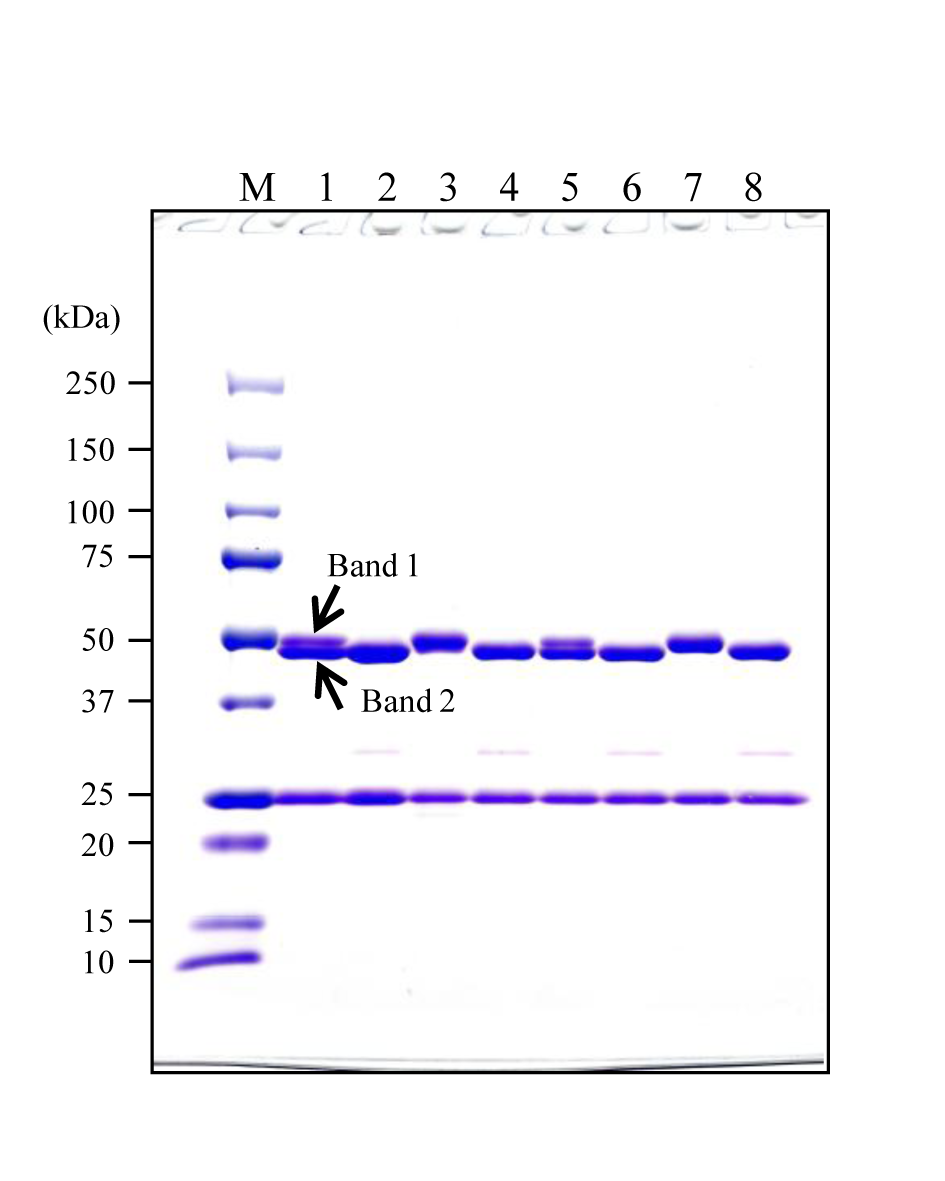

Supplement: S1 Fig — The fucosylated and nonfucosylated anti-CD20 IgG1 variants that were digested by PNGaseF were subjected to reducing 5–20% SDS-PAGE analyses. Lane M: molecular mass marker, lane 1: fucosylated Y296P without digestion, lane 2: fucosylated Y296P with digestion, lane 3: fucosylated wild-type without digestion, lane 4: fucosylated wild-type with digestion, lane 5: nonfucosylated Y296P without digestion, lane 6: nonfucosylated Y296P with digestion, lane 7: nonfucosylated wild-type without digestion, lane 8: nonfucosylated wild-type with digestion. (TIF) [file pone.0140120.s001.tif]

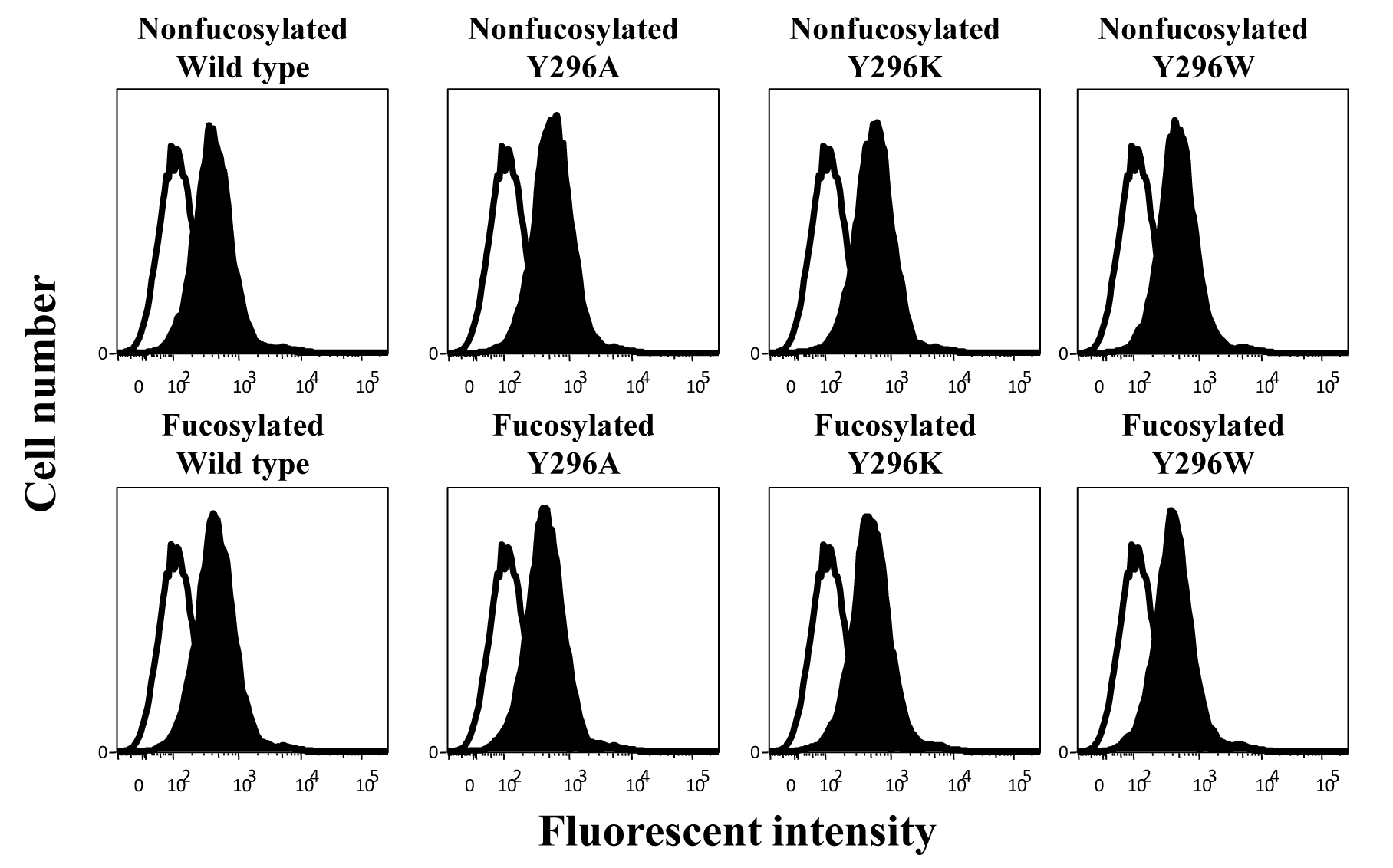

Supplement: S2 Fig — Rituximab variants binding to Raji cells were measured by flow cytometry. Cells were stained with 1 μg/mL anti-CD20 IgG1 rituximab variants (filled histograms) or staining buffer alone (blank histograms) at 4°C for 30 min, followed by staining with a detecting antibody (FITC-conjugated anti-human IgG antibody) at 4°C for 30 min. (TIF) [file pone.0140120.s002.tif]

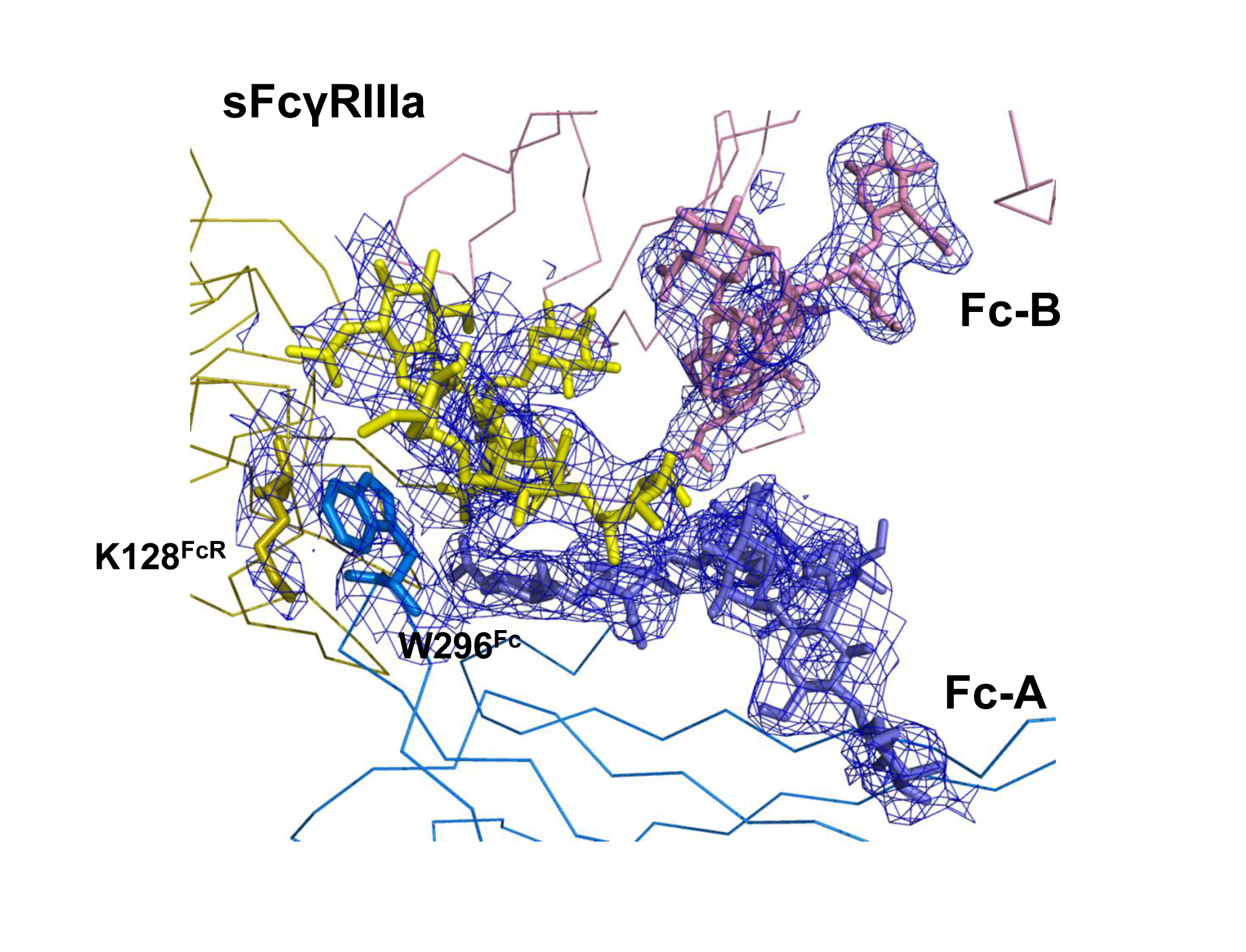

Supplement: S3 Fig — The F o-F c electron density map of N-glycans, Trp–296 (IgG1-Fc chain A), and Lys–128 (FcγRIIIa) contoured at 1.5 σ. (TIF) [file pone.0140120.s003.tif]
